# A simple 230 MHz photodetector based on exfoliated WSe_2_ multilayers†

**DOI:** 10.1039/d4lf00019f

**Published:** 2024-03-07

**Authors:** Fabian Strauß, Pia Kohlschreiber, Jakob Keck, Patrick Michel, Jonas Hiller, Alfred J. Meixner, Marcus Scheele

**Affiliations:** a Institute for Physical and Theoretical Chemistry, University of Tübingen 72076 Tübingen Germany marcus.scheele@uni-tuebingen.de; b Center for Light-Matter Interaction, Sensors and Analytics LISA+, University of Tübingen 72076 Tübingen Germany

## Abstract

We demonstrate 230 MHz photodetection and a switching energy of merely 27 fJ using WSe_2_ multilayers and a very simple device architecture. This improvement over previous, slower WSe_2_ devices is enabled by systematically reducing the RC constant of devices through decreasing the photoresistance and capacitance. In contrast to MoS_2_, reducing the WSe_2_ thickness toward a monolayer only weakly decreases the response time, highlighting that ultrafast photodetection is also possible with atomically thin WSe_2_. Our work provides new insights into the temporal limits of pure transition metal dichalcogenide photodetectors and suggests that gigahertz photodetection with these materials should be feasible.

## Abstract 2

We demonstrate 230 MHz photodetection and a switching energy of merely 27 fJ using WSe_2_ multilayers and a very simple device architecture.

## Graphical abstract

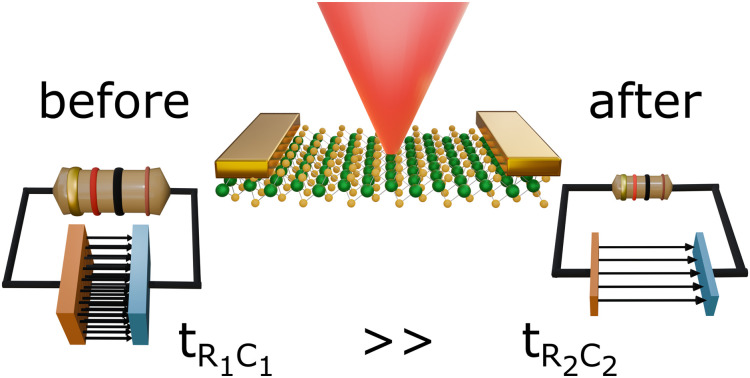

## Introduction

Next generation photodetectors have to meet several requirements to overcome the current limitations of silicon-based devices.^1–3^ They must be cheap, reliable in fabrication and exhibit low power consumption, for which high speed and low dark currents are essential.^2,4^ Transition metal dichalcogenides (TMDCs) are promising in this regard,^1–3^ especially since the increasing quality of flakes produced *via* chemical vapor deposition is closing the gap to the excellent properties of mechanically exfoliated ones, allowing reliable and relatively inexpensive production.^5–7^ However, achieving high switching speeds toward gigahertz photodetection remains challenging, in particular without compromising the responsivity too much. This is illustrated by the gain-bandwidth product, accounting for the necessity for a high gain/responsivity to have a long lifetime and thus a low bandwidth.^3,4,8,9^ The intrinsic response, *i.e.*, the pure material-based upper limit for photodetection without limitations such as the RC time of the device, has been shown to be in the picosecond regime.^10–12^ In contrast, regarding the application-relevant extrinsic response time, most reports have revealed response times of milliseconds to microseconds.^9,13–22^ Some groups have reported nanosecond response times,^23–26^ and in combination with highly advanced photonic circuits, even faster detectors are possible.^27–29^ The problem with such sophisticated fabrication techniques remains the scalability. Furthermore, many approaches are based on TMDC heterostructures^11,19,30^ or combinations of TMDCs with other materials,^31^*i.e.*, hBN,^9,19,22^ graphene^20,21^ or quantum dots,^32,33^ which in turn complicates fabrication.^2^

In this work, we study highly simple TMDC photodetectors, comprising only exfoliated multilayers or bilayers of pure WSe_2_ and gold top-contacts. We show that multilayer devices are RC limited and that reducing their photo resistance as well as the device capacitance affords a response time below 2 ns and an electrical bandwidth in excess of 230 MHz, which is unprecedented for pure TMDC photodetectors to our knowledge. The devices are operated at zero bias, leading to a switching energy of only 27 fJ per bit, highlighting the potential of TMDC photodetectors for low-power optical communication. We find response times <20 ns for bilayers, indicating that the deleterious persistent photocurrent known for MoS_2_ mono- and bilayers is not an issue for WSe_2_.

## Experimental section

### Fabrication

WSe_2_ photodetectors were fabricated following a standard scotch tape exfoliation technique^34^ onto HMDS-functionalised glass substrates. The multilayer devices show thicknesses between 5 and 32 nm. Once exfoliated, the contacts were patterned using optical photolithography with a maskless aligner (μMLA, Heidelberg Instruments). For geometries with channel lengths of less than 2 μm, electrodes were written using electron beam lithography (JEOL JSM-6500F). The metal contacts were evaporated with a thickness of 2.5 nm titanium followed by 10 nm of gold at a pressure of <2 × 10^−6^ mbar. The storage and examination were performed under atmospheric conditions.

### Transient photoresponse

The transient photoresponse was analysed using two different setups. First, on a Lake Shore Cryotronics CRX-6.5K probe station, described in more detail in our previous work^35^ and second, on a custom-built confocal microscope to enable diffraction limited illumination,^36^*cf.* Fig. SI15† for more details. In short, laser illumination was carried out with a square pulse laser (635 nm) switched on and off by a Hewlett Packard 33120A arbitrary waveform generator triggering a FSL500, PicoQuant laser driver to record the steady state response. To measure the impulse response of the sample, a pulsed laser, emitting pulses with a pulse length <500 ps at a repetition rate of 1 MHz (average output power 81 μW, 636 nm) controlled by a Taiko PDL M1 (PicoQuant) driver, was used. The square pulse laser had a nominal laser rise/fall time of less than 0.5 ns and an output power of approximately 2 mW at the fibre end face. The laser power was further reduced due to coupling losses from fibre to fibre in the case of the probe station setup or *via* coupling through pinholes, reflection in mirrors and beam splitters when coupled with the confocal microscope. The dark current and ON/OFF electrical measurements were performed with a Keithley instruments 2636B source meter. For time-resolved measurements, a Zurich Instruments UHF Lock-In amplifier was used with a Periodic Waveform Analyzer function averaging over 2 G samples in combination with a transimpedance amplifier (FEMTO DHPCA-100) when necessary. The electrode pads were connected with 50 Ω-matched tungsten probes and coaxial cables with bandwidths exceeding 1 GHz at the probe station. In the confocal setup, gold-plated probe tips and triaxial probe holders (79-8000-T-03 Micromanipulator) were used to make contact beneath a custom built faraday cage. For connection between triaxial cables and the BNC-input at the lock-in amplifier, a triax(F)-to-BNC(M) connector (Pomona) was used; however, manufacturer's bandwidth specifications were not provided. The bandwidths of other devices are 600 MHz for the lock-in amplifier and 175 MHz for the transimpedance amplifier at an amplification of 10^3^.

## Results and discussion

### Reduction of the photoresistance

**Fig. 1:**
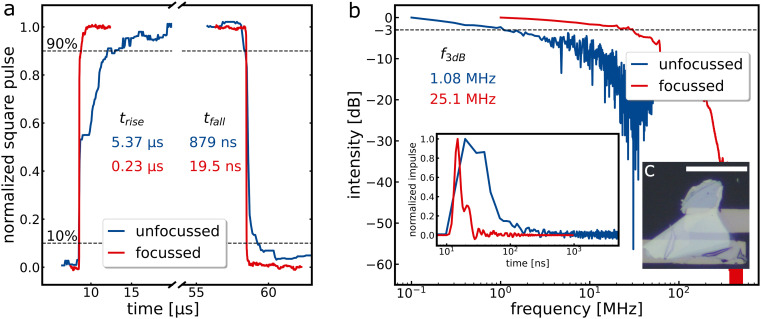
(a) Square pulse measurements of the same WSe_2_ flake characterised with unfocussed (blue) and focussed (red) 635 nm laser illumination and 0.5 V bias. The unfocussed measurement is conducted at 10 kHz, whereas the focussed one has a repetition rate of 100 kHz. The red trace is split and shifted horizontally to match the unfocussed curve for better visibility. The irradiance is 0.4 W cm^−2^ (unfocussed) over the whole channel and approximately 200 W cm^−2^ (focussed), with only a fraction of the channel width illuminated. (b) Power spectra of the 636 nm impulse laser with 100 kHz (blue) and 1 MHz (red). The inset shows the measurements in real-time. (c) Light microscopy images of WSe_2_ flakes fabricated with optical lithography. The scale bar is 20 μm.

Steady state and non-steady state measurements were performed to characterise the switching behaviour of the photodetectors. Typical illumination in the literature ranges from minimal laser powers in nW and sub-nW regimes up to irradiances of more than 10 kW cm^−2^.^9,13,16–23,30,31,37,38^ We begin by examining the photoresponse of a bulk WSe_2_ flake, Fig. 1c, under wide field illumination with an unfocussed laser beam illuminating an area of about 1.5 mm^2^ under ambient conditions (see Fig. SI1 and SI2† for dark current and optical microscopy images, “flake 1”). Typical steady-state and non-steady-state responses are shown in Fig. 1a (blue) and Fig. 1b (blue), respectively, which are consistent with our previous studies on WSe_2_.^39^ As the response time depends, among other things, on the photoresistance of the sample, we increased the irradiance per area by repeating same measurements within a confocal microscope using a diffraction-limited focussed laser beam. Considering the reduction of the illuminated area, as well as the coupling losses at the pinholes and beam splitters, this increases the irradiance from roughly 0.4 to 400 W cm^−2^. Additionally, the illumination position can be precisely controlled in the confocal setup, and the intensity can be varied by neutral density filters. By maximising the illumination intensity per area, the photoresistance in the steady state measurements decreases by a factor of 23, from 82.7 MΩ to 3.6 MΩ, leading to a greatly reduced response time (measured with a current amplifier), as shown in red in Fig. 1a. This behaviour indicates an RC limitation of the device.

The impulse response (*f*(*t*)) is fast Fourier transformed (FFT) to obtain the power spectrum (*P*(*ω*)): *P*(*ω*) = |FFT(*f*(*t*))|^2^. After conversion to the dB scale, *via* dB = 10 log_10_((*P*(*ω*))/*P*_1_) with the steady state power *P*_1_, 3 dB bandwidth, *i.e.*, the frequency at which the power drops to half its value, can be read out.^40^ Comparing two values for unfocussed and focussed measurements again reflects the factor 23, showing the dependence on the photoresistance and supports the hypothesis of RC-limitation. The non-normalised square pulse measurements can be seen in Fig. SI4.† ON/OFF measurements performed with the same sample after six months (Fig. SI3†) reveal ratios >10^4^ and long-term stability under ambient conditions.

**Fig. 2:**
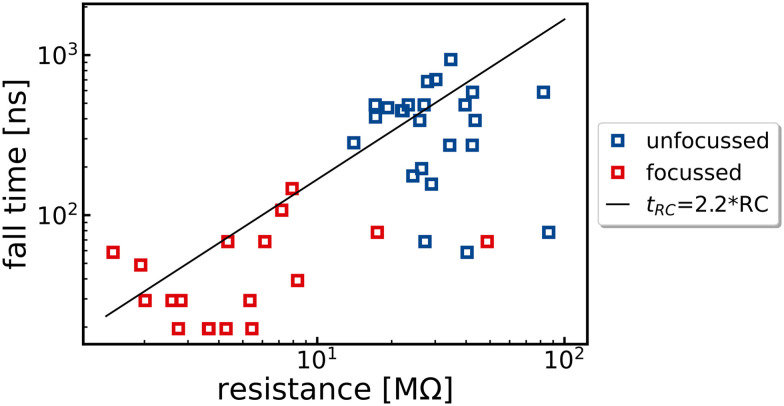
Fall time *vs.* photo resistance for all voltages. The data were obtained from various measurements performed at different laser intensities and positions on an optical lithography processed flake. The black line represents the calculated RC-limited fall time using the estimated capacitance of 7.6 fF for the used geometry, according to *t*_RC_ = 2.2 × RC.

To determine the influence of the photoresistance on the response time, the device was measured under different illumination intensities and positions of the laser focus on the sample. All measurements obtained in this way for the same device are summarized in Fig. 2. If the hypothesis of an RC limitation is correct, the slope of the linear fit to this data should resemble the capacitance of the device. The expected capacitance is obtained following Nabetl *et al.*:^41^*C* = *L*(*N* − 1)*ε*_0_(1 + *ε*_r_)(*K*(*k*))/(*K*(*k*′)) with the channel width *L* = 25 μm, the number of fingers *N* = 2, the vacuum permittivity (*ε*_0_), the dielectric constant of WSe_2_ (*ε*_r_ = 20, as in our previous studies^39^) and *K*(*k*) being the complete first order elliptical integral with *k* = cos(π/2(1 − *w*/(*w* + *g*))) and 
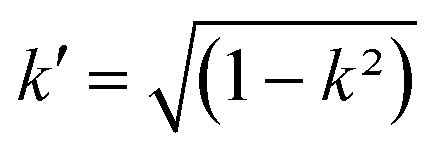
. The width *w* of the electrodes is 10 μm in the optical lithography structure, and the gap *g* between the two electrodes is 2.5 μm. This leads to a calculated capacitance of 7.6 fF. Multiplying by a factor of 2.2, which accounts for 10 to 90% rise/fall time values and affords the black line in Fig. 2, provides a reasonable fit to experimental data, thus strongly supporting the hypothesis of RC limitation.

### Reduction of the capacitance

We then aimed to further increase the speed of photodetection with multilayer WSe_2_ by reducing the device dimensions, and thus, the capacitance. With reference to parameters in the formula for the capacitance by Nabetl *et al.*, we reduce the electrode width (*w*) from 10 to 1 μm, the channel length (*g*) from 2.5 to 1 μm, and the channel width (*L*) from 25 μm to 20 μm; see Fig. SI5† for a scheme of the geometrical parameters. This decreases the expected capacitance from 7.6 fF to 4 fF.

**Fig. 3:**
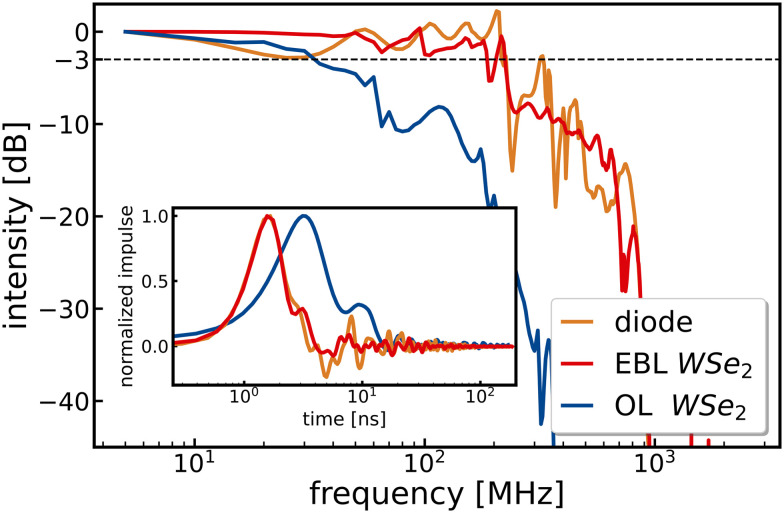
Power spectrum of a commercial photodiode (ochre) and an EBL fabricated WSe_2_ flake (red) in comparison with an optical lithography WSe_2_ flake (blue). The inset shows the impulse measurements in real-time. Both measurements were performed with a 636 nm pulsed laser excitation at 5 MHz. For the WSe_2_ detector, a bias voltage of 0.5 V is applied, and for the diode 5 V in the reverse bias direction. Irradiance for the measurements is approximately 400 W cm^−2^. Measurements of the diode and EBL WSe_2_ flake were performed without a transimpedance amplifier.

An image of this new device based on a multilayered WSe_2_ bulk flake is shown in Fig. SI1b† (“flake 2”), and the dark current as well as ON/OFF ratios are displayed in Fig. SI6–8.† At zero bias and 2 μW illumination at 635 nm, we find a photocurrent higher than 20 nA, as shown in Fig. SI8,† hinting at the presence of a built-in electric field presumably either due to slight height differences of the flake within the channel^42^ or to altered electric contacting of the electrodes as a result of the electron beam evaporation process.^43^Fig. 3 displays the non-steady state response and power spectrum of the new device (red curve) with a fall time <2 ns and a 3 dB bandwidth of 230 MHz. For comparison, the photo response curve of the previous device from Fig. 1 is also displayed (blue curve) to illustrate the effect of the reduced capacitance. In addition, we measure the power spectrum of a commercial photodiode with a nominal fall time of 200 ps (ochre curve) and expected 3 dB bandwidth of 1.75 GHz to find essentially the same 230 MHz cut-off as with the improved WSe_2_ device. This strongly suggests that the measurements are limited by the setup, presumably due to the applied cables and connectors, the 600 MHz low pass filter within the lock-in amplifier, and the fact that the true speed of the improved WSe_2_ device might be even faster. Further evidence for such limitations is found in periodic wiggles in the non-steady state response and resulting noisy power spectrum, which can be attributed to reflections inside the cables as detailed in the ESI,† Fig. SI13.

We note that neither changing the bias between 0 and 0.5 V (Fig. SI9†) nor altering the laser intensity with optical density filters between 0.08 and 0.54 (Fig. SI14†) has a significant effect on the 3 dB bandwidth, again suggesting that all of these measurements fall into the setup limit. In principle, further reduction of the channel length below 1 μm (Fig. S11†) and channel width to decrease the resistance and capacitance, respectively, could be easily implemented with standard electron beam lithography. For instance, an easily feasible reduction of the channel width from 20 μm to 1 μm would decrease the capacitance by a factor of 20, *cf.* Fig. SI10.† However, we neglect such further optimisations at this point due to the speed-limitations of our setup.

For low-power optical communication, the switching energy is an important device parameter. For a measurement with zero bias, there is no additional energy cost for the applied voltage and switching energy amounts to <27 fJ per bit upon dividing a typical incident laser power of 6.25 μW by the (setup limited) 230 MHz bandwidth. Using a square pulse laser with comparable output power (2 μW) and irradiance (100 W cm^−2^), we calculate the responsivity of the device under typical operating conditions of 50 mA W^−1^ using ON/OFF characteristics in Fig. SI7.† We note a strong power-dependence of the responsivity with quickly declining values at higher powers in line with earlier reports.^23,30^ Under the assumption that the dark current dominates the noise, we calculate the specific detectivity according to:^25^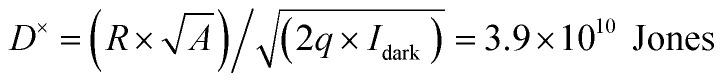, with 50 mA W^−1^ for the responsivity, an area *A* of 20 μm^2^, the electron charge *q* and a dark current of 1 pA. We obtain the external quantum efficiency as EQE = (*R* × *hν*)/*q* with the responsivity, Planck's constant, frequency and electron charge, respectively. With a responsivity of 50 mA W^−1^ at a wavelength of 636 nm, this yields a value of approximately 10% which is in good agreement with earlier reports.^23^

### Reduction of the flake thickness to bilayer

**Fig. 4:**
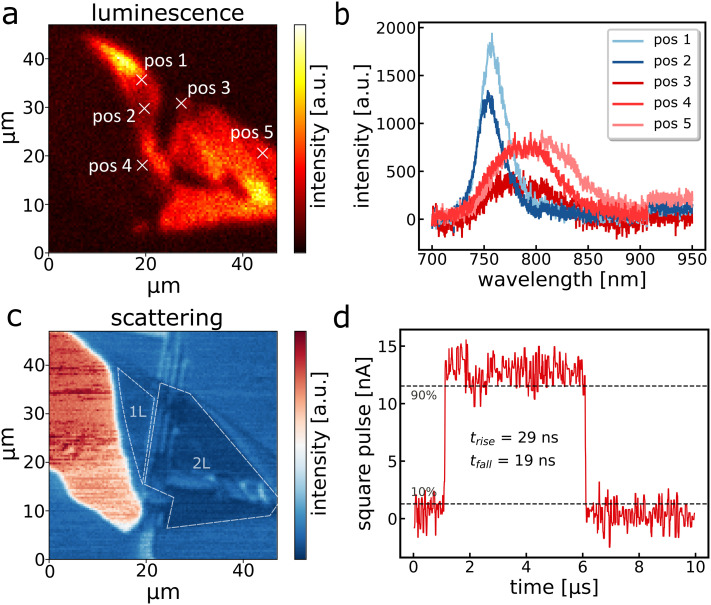
a) Luminescence scan of a mono-/bilayer WSe_2_ flake. b) Representative spectra recorded at positions marked in a). c) Scattering image of the same flake. The dashed white lines mark the positions of the mono- and bilayer. d) Steady state measurement with a 635 nm square pulse laser driven at 100 kHz in a confocal setup for a bilayer WSe_2_ flake.

For some photonic applications, it is desirable to decrease the thickness of TMDC devices from multilayers to bi- or even monolayers, *e.g.* to increase the photoluminescence quantum yield.^6^ However, for the most widely studied TMDC, MoS_2_, Tang *et al.* have revealed that the speed of photodetection decreases by several orders of magnitude when approaching *via* bi- or monolayer thickness.^44^ This is a result of persistent photocurrents^45^ due to interface trap states,^8^ which are very prominent in atomically thin MoS_2_ and provide a serious drawback for optical communication with MoS_2_ photodetectors. To assess whether similar drawbacks exist for WSe_2_, we have fabricated an ultra-thin WSe_2_ device using the same geometries as for the 230 MHz multilayer photodetector (see Fig. SI1c†). Fig. 4a displays a luminescence map of the flake with respective spectra shown in Fig. 4b. Positions 1 and 2 are the characteristic emission peaks of WSe_2_ monolayers at approximately 750 nm, whereas others show less intense and red-shifted bilayer emission.^46^ Based on the luminescence, the scattering in Fig. 4c and the optical image (Fig. SI1c†), we reconstructed the position of the mono- and bilayer as marked in Fig. 4c. From this, we infer that this photodetector consists exclusively of mono- and bilayers of WSe_2_ within the channel. While a reliable power spectrum cannot be obtained due to the relatively weak absorption and photocurrent signal, we obtain a fall time of 19 ns in response to a square pulse (Fig. 4d), demonstrating that the speed of WSe_2_ photodetectors is much more robust against surface trap states, in stark contrast to MoS_2_. We attribute the remaining speed difference compared to our champion multilayer WSe_2_ device to the higher photoresistance due to the weaker absorption, which increases the RC time.

## Summary and conclusion

We have systematically reduced the response speed of RC-limited, multilayered, pure WSe_2_ photodetectors toward a record-high 3 dB bandwidth of 230 MHz. We have shown that optical switching with this device can be carried out at zero bias, requiring just 27 fJ per switching event. Reducing the detector thickness to mono- and bilayers of WSe_2_ only weakly decreases the response speed, rendering WSe_2_ advantageous over MoS_2_ for fast optical communication. Further miniaturizations of the device geometry have the potential for gigahertz photodetection with such easily fabricated WSe_2_ photodetectors, which exhibit long-term stability under ambient conditions.

## Conflicts of interest

There are no conflicts to declare.

## Supplementary Material

LF-001-D4LF00019F-s001
