# Supplementary material for: A simple 230 MHz photodetector based on exfoliated WSe2 multilayers
Source: RSC Appl Interfaces. 2024 Mar 7;1(4):728–33. doi: 10.1039/d4lf00019f (PMC11231687; doi:10.1039/d4lf00019f)
Supplement: LF-001-D4LF00019F-s001 [file LF-001-D4LF00019F-s001.pdf]

Electronic Supplementary Material (ESI) for RSC Applied Interfaces.
This journal is © The Royal Society of Chemistry 2024

Supplementary Information:
A simple 230 MHz Photodetector Based on Exfoliated WSe_2 Multilayers

Fabian Strauß[1,2,a], Pia Kohlschreiber[1,2,a], Jakob Keck[1,2], Patrick Michel[1,2], Jonas Hiller[1], Alfred J. Meixner[1,2], Marcus Scheele[1,2,*]

[1] Institute for Physical and Theoretical Chemistry, University of Tübingen, 72076 Tübingen, Germany.

[2] Center for Light-Matter Interaction, Sensors and Analytics LISA+, University of Tübingen, 72076 Tübingen, Germany.

Light microscopy images and height profile of the flakes 1- 4

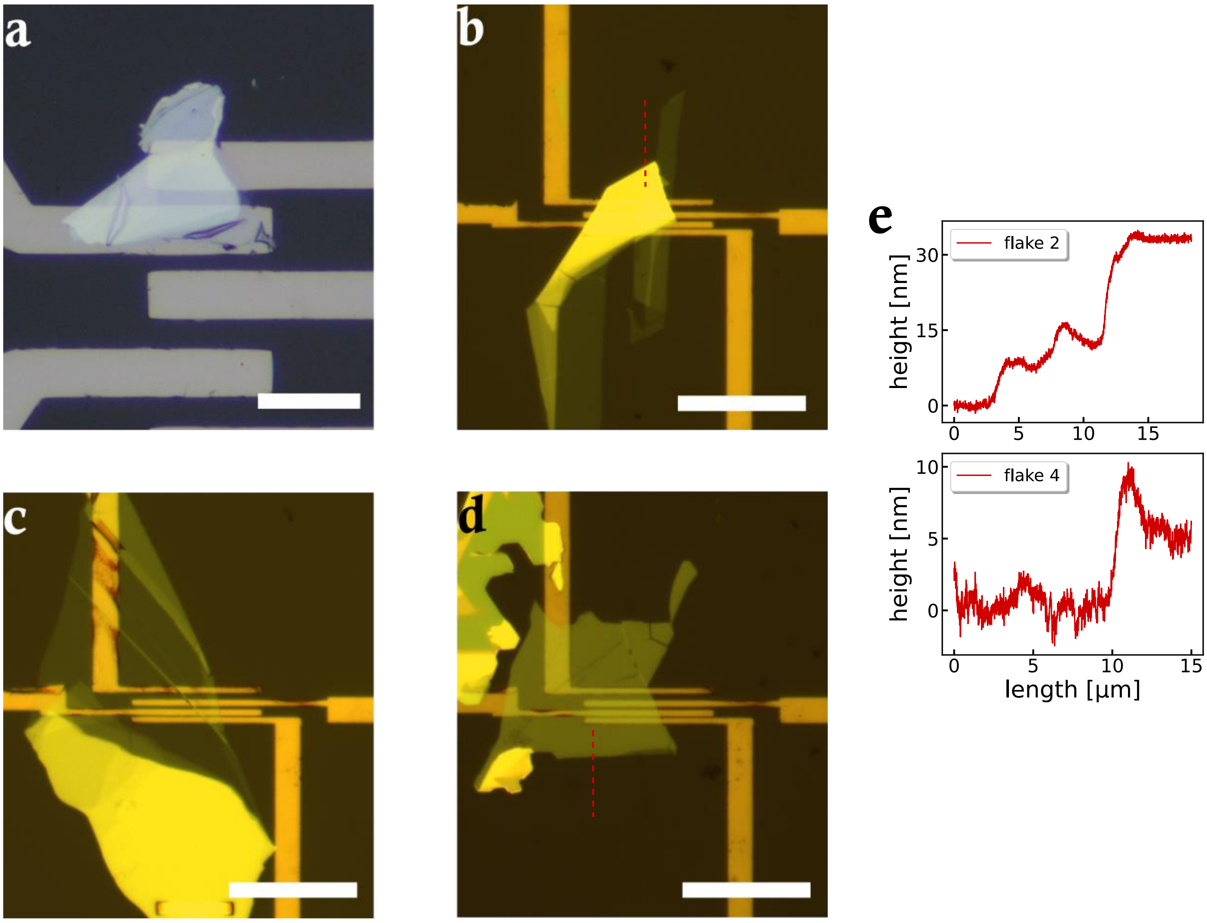

Figure SI1: Light microscopy images of the WSe_2 flakes measured for this work. a) shows the flake 1 which was fabricated with optical lithography. Flakes 2-4 are fabricated via electron beam lithography and shown in b)-d). The scalebar is 20 µm in each image. e) shows the height profile of flake 2 (b) and flake 4 (d). The lines, where the thickness were measured are shown as dashed lines in the microscopy images. Profiles are measured at a Bruker DektakXT.

This figure shows the thicknesses of the measured flakes. Flake 1, shown in (a) has a thickness between flake 2 (32 nm) and flake 4 (5 nm), shown in SI1e, as can be seen in the microscopy images. Flake 3 is further characterised with luminescence spectra, see Figure 4. It consists of a monolayer region, cf. positions 4 and 5 in Figure 4, and a bilayer region, including the electrodes.

Dark current flake 1

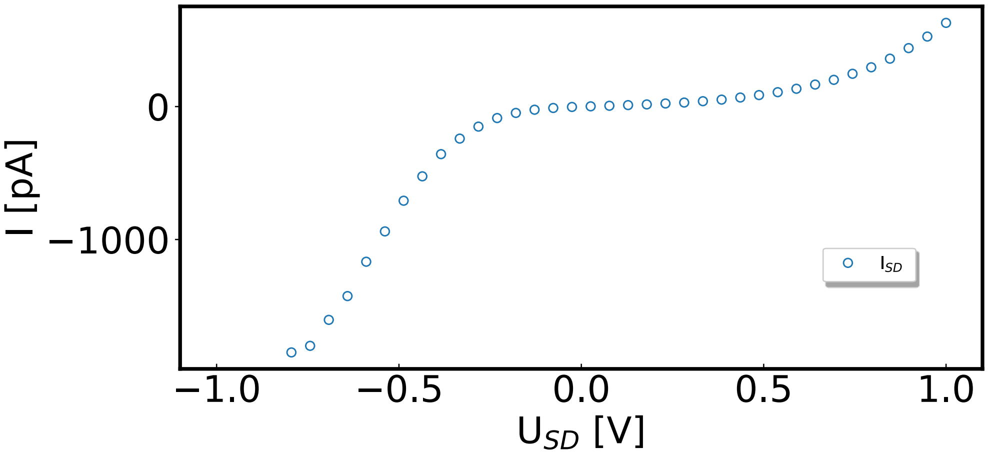

Figure SI2: Dark current of flake 1 from -1 to +1 V. The IV-curve shows a non-ohmic behaviour, typical for WSe_2.

In addition to the non-ohmic behaviour, the flake shows a slight asymmetry which can be caused by different heights.[1]

ON/OFF ratio of lake 1

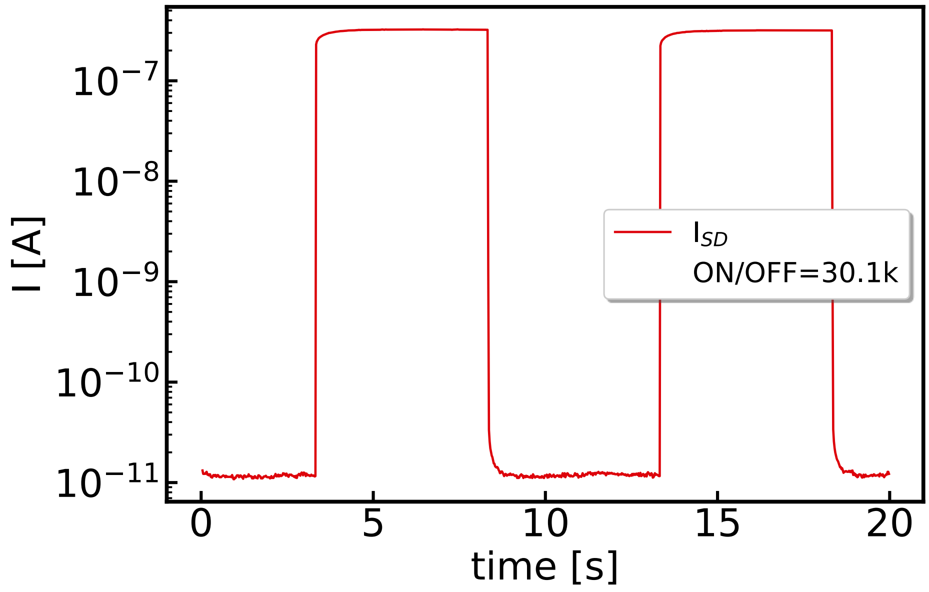

Figure SI3: ON/OFF ratio for flake 1 with 200 mV bias applied and 31 µW illumination power with a 635 nm laser driven at 0.1 Hz. With a widefield lens the whole channel was illuminated evenly.

The ON/OFF ratio exceeded the measurement range of the Keithley 2636B. Therefore, the lower values were set from e-10 to e-11, to reflect the enormous ON/OFF ratio more accurately. Compared to Figure SI2, the dark currents at 200 mV bias are still lower than displayed here.

Square pulse measurements – current height

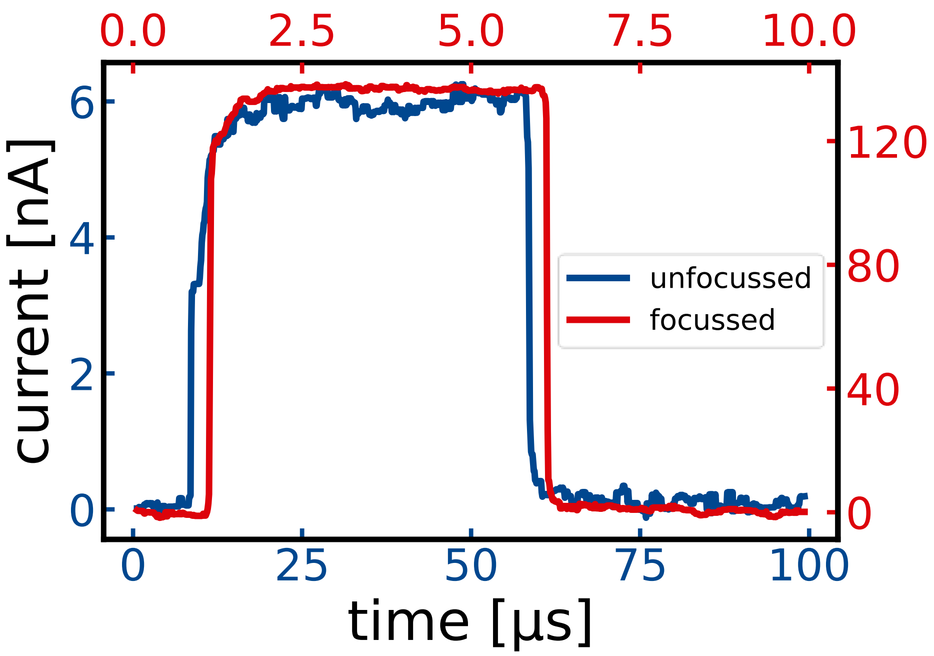

Figure SI4: Here, the exact same measurements like in Figure 1a are shown without normalization. The left y-axis and the lower x-axis correspond to the 10 kHz measurement without a focus. The red axis belongs to the 100 kHz measurement of the same flake under illumination. Irradiances are 0.4 W/cm² for the unfocussed measurement and 400 W/cm² for the focussed one.

Scheme of geometrical terminology

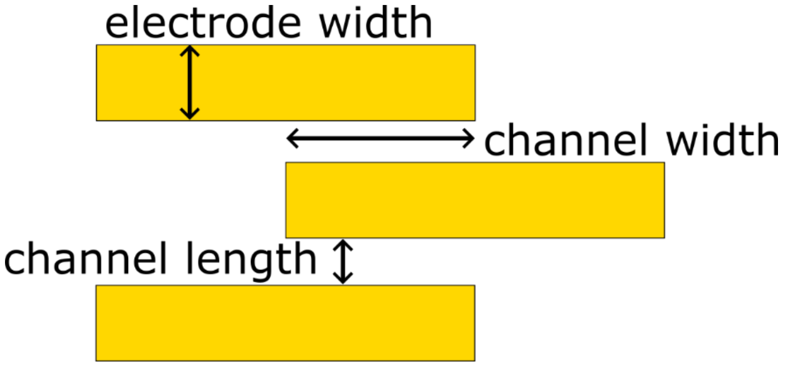

Figure SI5: Scheme of the contact geometry with specification of the used geometrical terminology.

Dark current flake 2

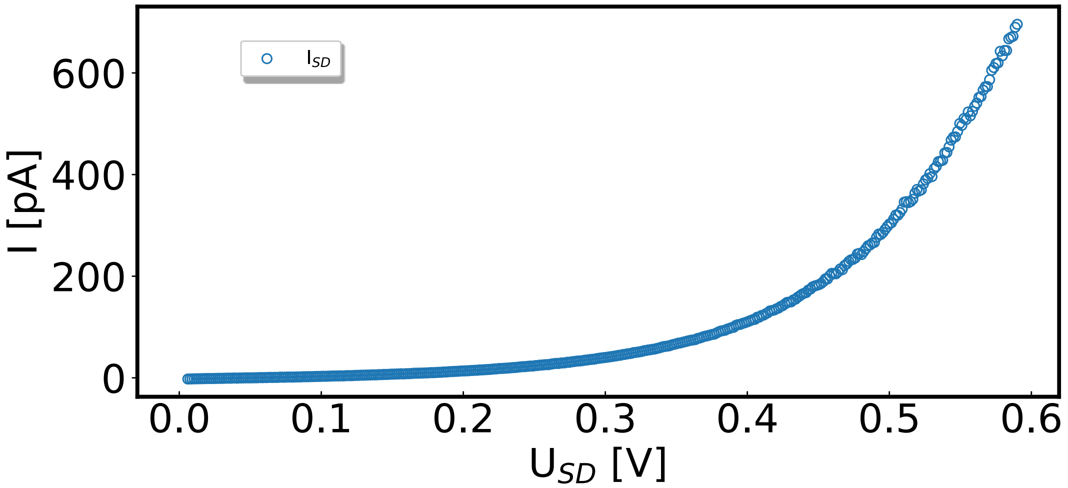

Figure SI6: Dark current of flake 2 from 0 to 0.6 V. The IV-curve shows a similar behaviour like before for flake 1.

ON/OFF ratio of flake 2

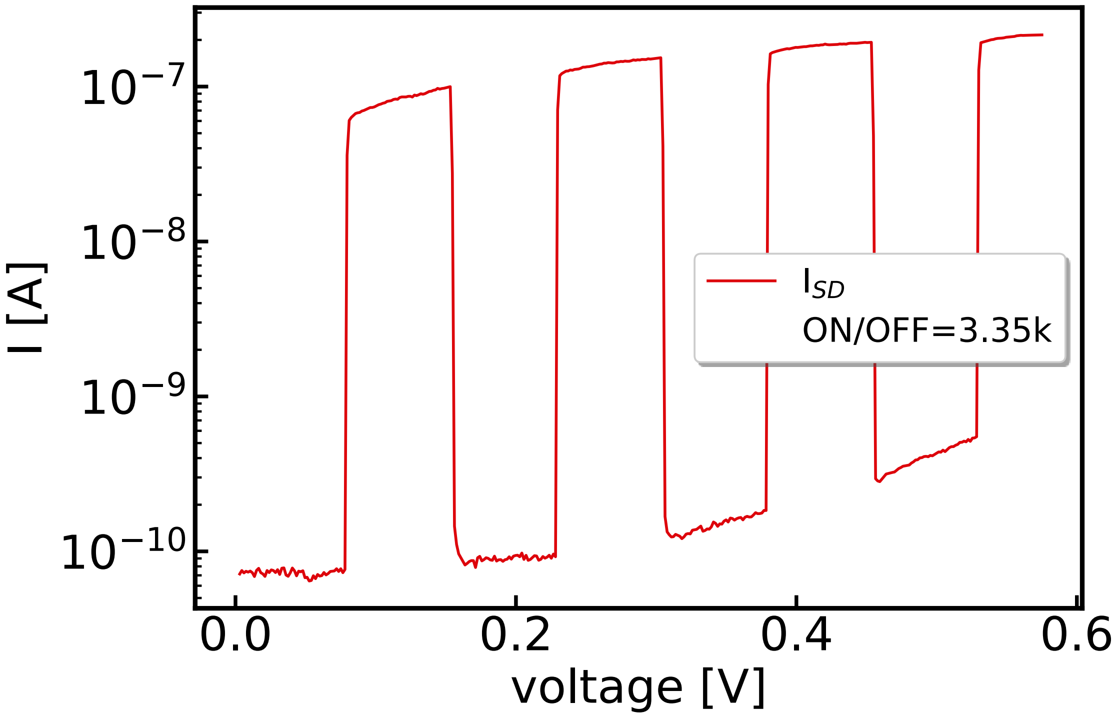

Figure SI7: ON/OFF ratio for flake 2 with a sweeping bias from 0 V to 0.6 V applied and 2 µW illumination power with a 635 nm laser driven at 0.1 Hz.

Figure SI7 shows the same dark current behaviour as before seen for Figure SI6. At the same time, the light current is increasing with higher voltage, but not as steep as the dark current. Thus, the ON/OFF ratio is shrinking for higher applied voltages and the displayed ON/OFF is the maximal ratio obtained.

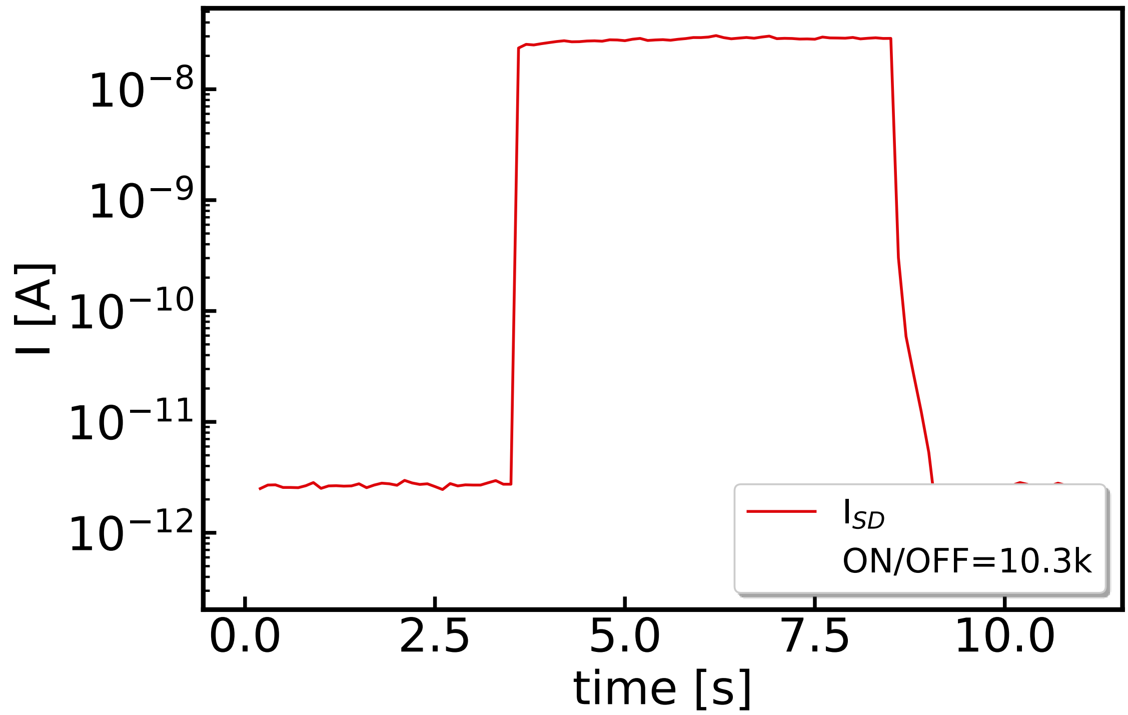

Figure SI8: ON/OFF ratio for flake 2 with a bias of 0 V and 2 µW illumination power with a 635 nm laser driven at 0.1 Hz. The focus size was approximately 2 µm.

Variation of bias for flake 2

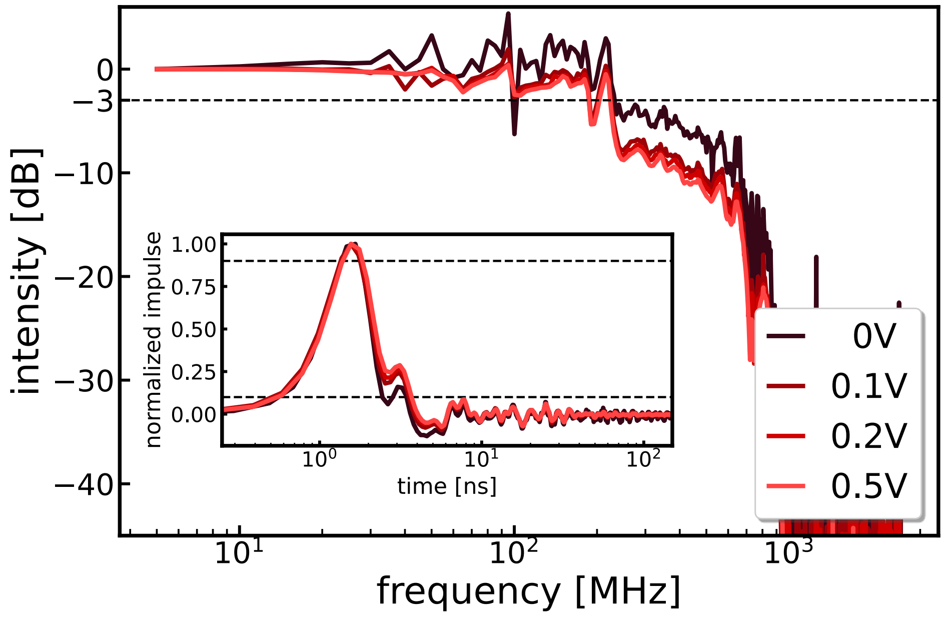

Figure SI9: Influence of different applied bias voltages for flake 2 on the power spectrum. The 636 nm impulse laser was driven at 5 MHz repetition frequency.

In Fig. SI9 the voltage is varied to check for a transit limitation of the 1 µm channel of flake 2. Since no difference between 0.1 V and 0.5 V is visible, no transit limitation is detectable at least within the 230 MHz setup limitation. The theoretical transit time can be estimated with: $t_{trans} = {d^2}/{\mu U} = {(1\mu\mathrm{m})^2}/{100\,{cm^2}/{Vs} * 0.1V} = 1ns$. Thereby, even the lowest voltage already yields a transit time of

1 ns with a moderate mobility estimated from few layer flakes.[2] Accordingly, higher voltages would only be even faster than this time.

Simulated Capacity

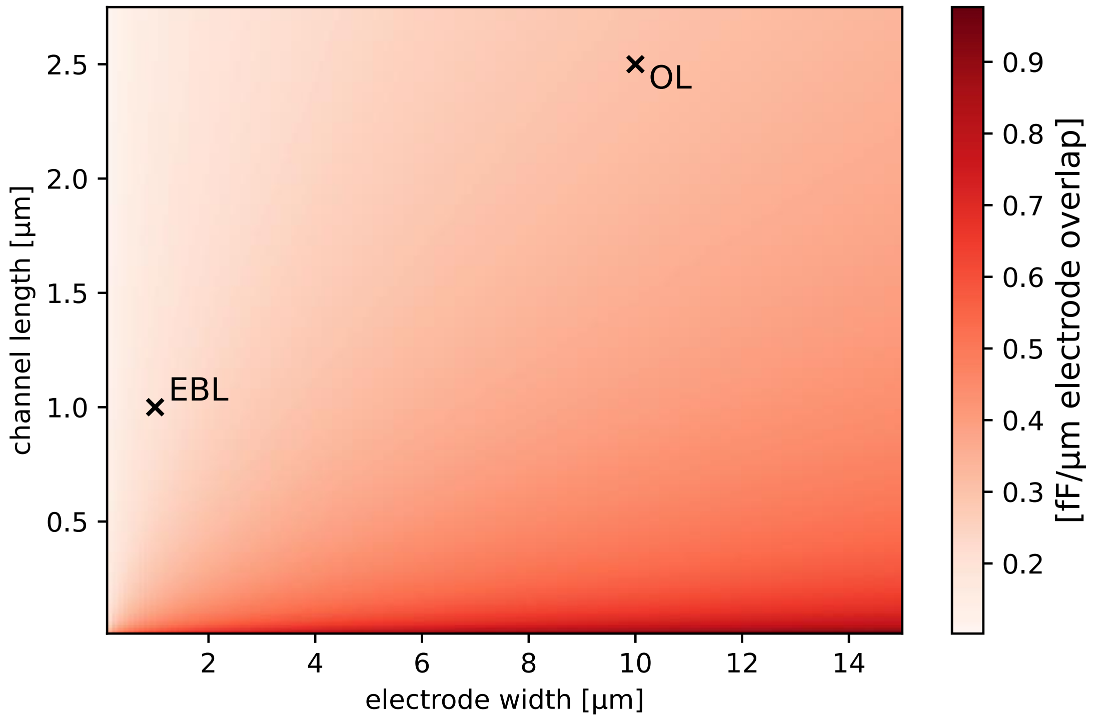

Figure SI10: Capacitance simulated for a variation of electrode width w and channel length g according to $C = L(N-1)\varepsilon_0(1+\varepsilon_r)\frac{K(k)}{K(k')}$ *with* $k = cos\left(\frac{\pi}{2}\left(1-\frac{w}{w+g}\right)\right)$ *and* $k' = \sqrt{1-k^2}$. ε_r *is set to 20, N to 2 and L is set to 1 µm. The crosses indicate the capacitances for the different lithographic approaches optical lithography (OL) and electron beam lithography (EBL).*

Figure SI10 shows the calculated capacitance in fF per µm electrode overlap. For the L = 25 µm used in optical lithography and L = 20 µm used in electron beam lithography in this work, the capacitance is 7.6 fF and 4 fF respectively. The trends that can be seen follow the intuition, that the capacitance increases for larger electrode widths, longer channel widths and shorter channel lengths, cf. Figure SI5 for clarification of the geometry.

Channel length and photo resistance vs fall time for flake 3 and 4

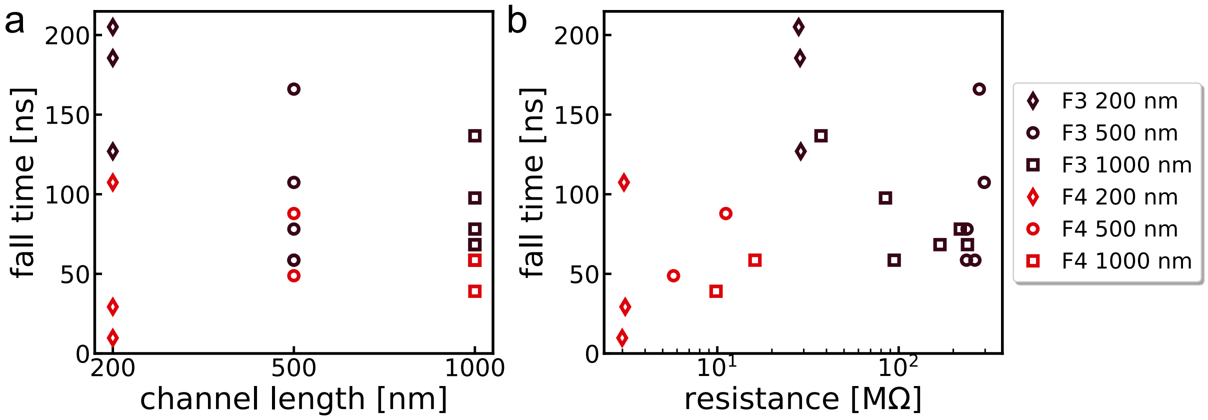

Figure SI11: a) channel length plotted versus fall time for the bilayer flake, flake 3 and the few layer flake, flake 4. b) the same measurements of a) are plotted in a resistance – fall time plot. All measurements are performed with a square pulse laser driven with 100 kHz and a laser power of 5.25 µW. The bias voltages are 1 V for 1 µm channels, 0.5 V for 500 nm and 0.2V for 200 nm channels.

Figure SI11 shows the variation of the channel length for the bilayer and the few layer flake. The idea behind a shortening of the electrode gap is again an acceleration of the detector due to the decrease of resistance, due to less material[3] and a shortening of the transit time[4]. For the two flakes shown here, it can be seen, that the shortening shows an ambiguous trend and the main limiting mechanism for the steady state measurements still seems to be the photoresistance, as can be seen in Figure SI10.

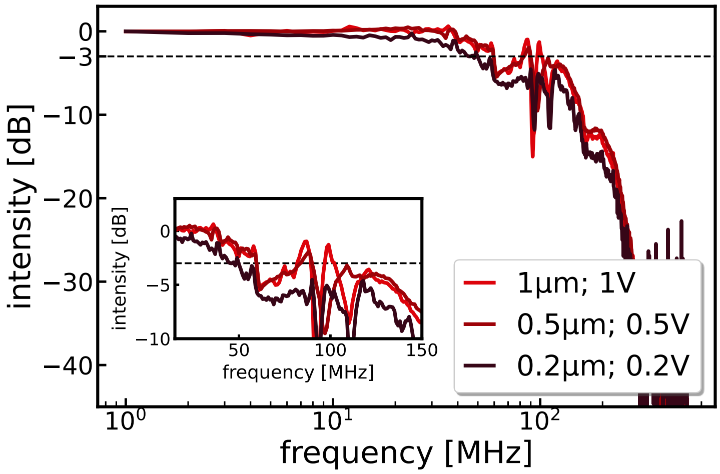

Figure SI12: Influence of the channel length on the power spectra of 1 MHz 636 nm laser measurements of flake 4. The bias is adjusted to get similar electric fields in the different channels.

The power spectrum tends to show a decreased bandwidth for the 200 nm channel. But all the bandwidths are close to the limit of our setup and thus too noisy, to observe a solid trend.

Cable reflections and influence on the Bandwidth

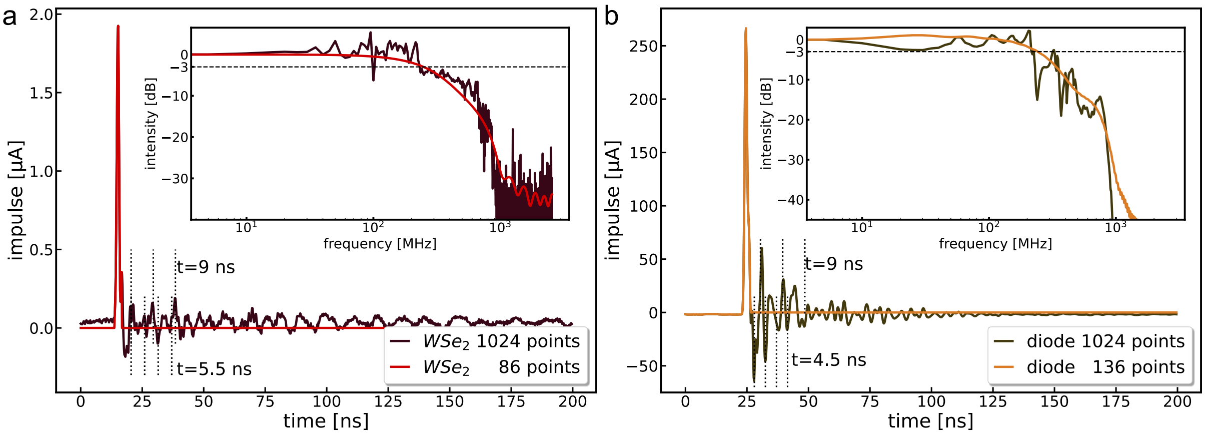

Figure SI13: Impulse and Fourier transformed impulse measurements for a) a WSe_2 sample (flake 2; 0 V) and b) a commercial Photodiode FDS015 (Thorlabs) with 200 ps nominal fall time. The dashed lines mark periodicities found in the time regime.

The impulse response decays very fast to zero followed by periodic signals. If the period of those signals is taken and multiplied with 2/3 times the speed of light *c*, which approximately is the speed of charges inside the cables, it can be attributed to reflections in the cables with a cable length of about 9 ns*2/3*c = 1.8 m which roughly agrees with the length of the cable we have used (1.7 m).

If the periodic signal following the delta pulse is set zero, then the power spectrum loses the spikes observed at frequencies larger than 40 MHz. Thereby, the frequency limit of our setup can estimated to be between 230 and 240 MHz from the measurement of the commercial diode with a nominal bandwidth of 1.75 GHz.

Variation of the illumination intensity for flake 2

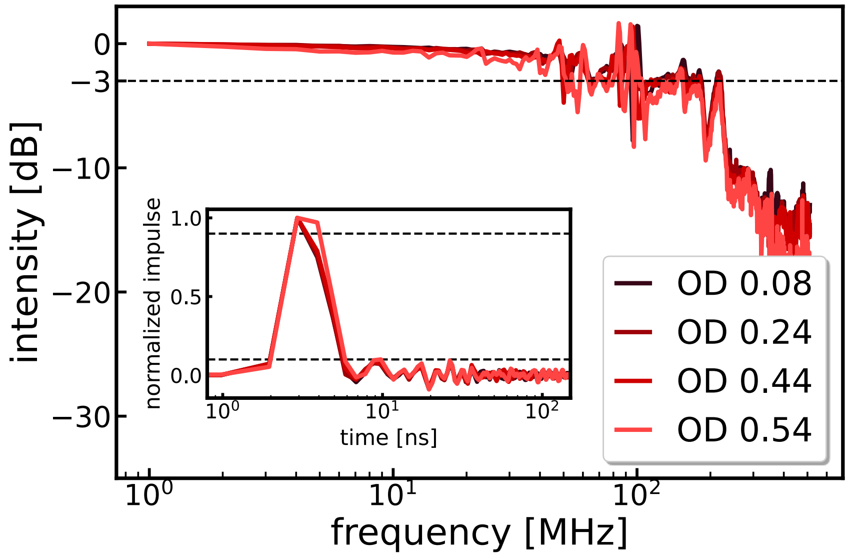

Figure SI14: Power spectra and impulse responses for 1 MHz 636 nm measurements of flake 2 for varied OD filters to alter the laser intensity to check for a RC limitation.

By varying the irradiation onto the sample, an RC limitation can be excluded or shown, since the RC-time is a limiting factor. In combination with Figure SI9, the variation of the transit time, the limiting extrinsic mechanisms for a photodetector can be observed. Here, no influence of the photoresistance onto the response speed can be observed, thus the detector is either not RC-limited or the setup limit hides any dependencies.

Scheme of the custom built confocal microscope

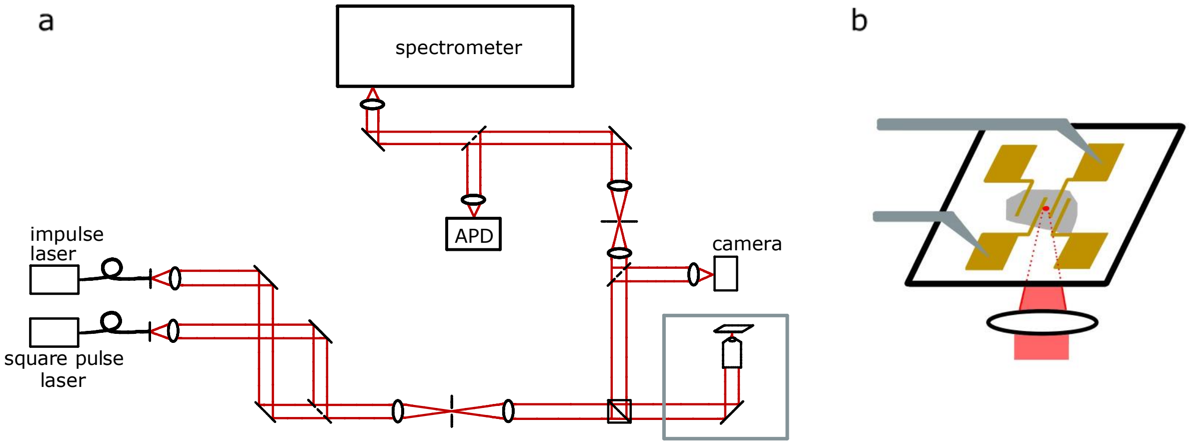

Figure SI15: a) Sketch of the confocal setup used for the focussed experiments. The part inside the grey box is rotated out of the plane and shown in more detail in (b).

Figure SI15 shows the free beam setup. In more detail: The laser beam diameter is expanded by a telescope containing a 30 µm pinhole. The light is focused onto the sample through a *Spindler und Hoyer* 20x NA 0,5 objective after passing through an 50/50 non polarizing beam-splitter. For spectra collection and confocal imaging, the emission light is collected via the beam-splitter and send through the detection telescope with an inserted 100 µm pinhole. The signal is collected on a ProEM+ 512B eXcelon camera attached to a Princeton Instruments Acton SP-2-500i spectrometer and a PerkinElmer optoelectronics SPCM AQR-13 (APD) respectively.

The minimal focal spot size can be estimated by the diameter of the Airy disc as follows:

$$D_{Airy} = 1.22 \cdot \frac{\lambda}{NA} = 1.22 \cdot \frac{636nm}{0.5} = 1.55\ \mu\text{m}$$

References

(1) Gao, W.; Zhang, S.; Zhang, F.; Wen, P.; Zhang, L.; Sun, Y.; Chen, H.; Zheng, Z.; Yang, M.; Luo, D.; Huo, N.; Li, J. 2D WS_2 Based Asymmetric Schottky Photodetector with High Performance. *Adv. Electron. Mater.* **2021**, *7*, 2000964.

(2) Zhou, H.; Wang, C.; Shaw, J. C.; Cheng, R.; Chen, Y.; Huang, X.; Liu, Y.; Weiss, N. O.; Lin, Z.; Huang, Y.; Duan, X. Large Area Growth and Electrical Properties of P-Type WSe_2 Atomic Layers. *Nano Lett.* **2015**, *15*, 709–713.

(3) Schedel, C.; Strauß, F.; Kumar, K.; Maier, A.; Wurst, K. M.; Michel, P.; Scheele, M. Substrate Effects on the Bandwidth of CdSe Quantum Dot Photodetectors. *ACS Appl. Mater. Interfaces* **2021**, *13*, 47954–47961.

(4) Sorger, V. J.; Maiti, R. Roadmap for Gain-Bandwidth-Product Enhanced Photodetectors: Opinion. *Opt. Mater. Express* **2020**, *10*, 2192.
